# Geographic Divergence in Heat Tolerance and Cross-Generational Responses of the Invasive Mealybug *Dysmicoccus neobrevipes*

**DOI:** 10.3390/insects17030328

**Published:** 2026-03-17

**Authors:** Yusha Wang, Dewei Li, Huiwen Huang, Andrew G. S. Cuthbertson, Zhongshi Zhou, Zhenqiang Qin

**Affiliations:** 1 Institute of Plant Protection, Guangxi Academy of Agricultural Sciences, Nanning 530007, China; yusha_wang@126.com (Y.W.); ldw11023@163.com (D.L.); hhwyx163@163.com (H.H.); 2 State Key Laboratory for Biology of Plant Diseases and Insect Pests, Institute of Plant Protection, Chinese Academy of Agricultural Sciences, Beijing 100193, China; 3 National Nanfan Research Institute, Chinese Academy of Agricultural Sciences, Sanya 572019, China; 4 Independent Researcher, York YO10 5AQ, UK; andrew_cuthbertson@live.co.uk

**Keywords:** thermal adaptation, population divergence, cross-generational effects, pest

## Abstract

As a result of global climate change, insects are increasingly being exposed to extreme temperature events; yet population-level variation in heat tolerance and its underlying mechanisms remain poorly understood. In this study, we investigated thermal adaptation in four geographically distinct populations of the invasive mealybug *Dysmicoccus neobrevipes* from southern China. The populations were subjected to acute heat stress across a gradient of temperatures where survival, fecundity, offspring viability, and sex ratio were quantified. We found pronounced geographic divergence in upper thermal limits: populations from warmer regions (Guangdong and Hainan) exhibited better survival, more stable reproductive output, and greater tolerance in offspring compared with populations from cooler regions (Guangxi and Yunnan). Thermal responses followed a nonlinear pattern, with moderate heat often enhancing performance, while temperatures above physiological thresholds triggered abrupt declines. Under heat stress, life-history strategies differed among populations, with some exhibiting stress-induced reproductive investment and others showing vulnerability across all traits. Importantly, acute heat exposure produced cross-generational effects, highlighting that parental thermal history can influence offspring performance. These results demonstrate that population-specific climatic adaptation, nonlinear physiological limits, and life-history trade-offs jointly shape thermal tolerance. Understanding these mechanisms provides a predictive framework for anticipating invasive pest expansion under future climatic warming and informs region-specific pest management strategy development.

## Simple Summary

Understanding how insects cope with extreme heat is essential due to continual global warming. Here, we examined four local geographic populations of the invasive mealybug *Dysmicoccus neobrevipes* from southern China and compared their responses to acute high-temperature stress. Under acute high-temperature stress, populations from warmer regions (Guangdong and Hainan) showed higher heat tolerance, better survival, and more stable reproduction than those from cooler areas (Guangxi and Yunnan). Moderate heat sometimes improved survival and reproductive performance, but temperatures beyond physiological limits caused rapid declines in both traits. Heat stress also affected the next generation, indicating that thermal conditions experienced by parents influenced offspring performance. These findings demonstrate that local climate conditions shape population-level thermal adaptation and that heat tolerance involves nonlinear physiological limits and life-history trade-offs. The results aid in predicting future population expansion under global warming and provide guidance for region-specific pest management strategy development.

## 1. Introduction

Global climate change has become one of the most significant ecological challenges of the current age. It fundamentally alters species interactions, ecosystem functioning, and agricultural stability [1,2]. Rising temperatures, particularly the increasing frequency of extreme heat events, exert strong physiological and demographic constraints on ectotherms. In insects, temperature regulates metabolic rate, development, reproduction, and population persistence [3], and exposure to temperatures beyond upper thermal limits can disrupt cellular homeostasis, reduce fecundity, impair development, and even trigger population collapse [4,5,6]. Conversely, moderate warming within the optimal activity-season temperature range may enhance physiological performance, accelerate development, and facilitate population growth [3,7]. Together, these contrasting outcomes underscore the importance of understanding how insect populations respond and adapt to spatially heterogeneous thermal environments that include both routine activity-season temperatures and episodic heat extremes.

Thermal adaptation in insects is shaped by two ecologically distinct but complementary components of environmental temperature: (i) the routine thermal conditions experienced during their main activity season and (ii) episodic extreme heat events that impose acute physiological stress [7,8,9]. Thermal performance theory predicts that populations reach maximal fitness within a characteristic temperature range determined by local climatic regimes [10,11]. Extreme heat events, in contrast, primarily define upper thermal limits and survival under acute stress [8,9,12]. Such events can also induce adaptive physiological adjustments, including elevated critical thermal maxima (CT_max_), enhanced cuticular hydrocarbon protection, and increased heat-shock protein expression that collectively increase resilience to thermal stress [13,14]. As natural populations experience diverse and spatially structured temperature regimes, they are exposed to heterogeneous selective pressures. This results in geographically patterned thermal variation that can drive pronounced divergence in thermal physiology, performance, and tolerance among populations, thereby shaping demographic responses and adaptive potential under environmental change [3,13,15,16,17].

*Dysmicoccus neobrevipes* Beardsley, 1959 (Hemiptera: Pseudococcidae) is an invasive mealybug with expanding global distribution and a substantial agricultural impact, particularly on its primary host, sisal (Asparagaceae; *Agave sisalana* Perrine) [18,19]. Laboratory studies indicate that the optimal developmental and reproductive temperature range of this species lies between 23 and 29 °C [20]. In subtropical regions, seasonal variation in temperature is therefore likely to play a key role in shaping the activity and population dynamics of invasive mealybugs, potentially creating cooler periods that are particularly important for population persistence and outbreak risk. During these cooler seasons, monthly mean temperatures are approximately 18 to 23 °C in Guangxi province (GX), 19 to 23 °C in Yunnan province (YN), 21 to 25 °C in Hainan province (HN), and 20 to 24 °C in Guangdong province (GD), according to long-term climate records from the China Science and Technology Resources Sharing Network (http://data.cma.cn) (accessed on 9 January 2025). These temperature ranges broadly overlap with the species’ reported thermal performance optimum [20]. In summer, maximum daily temperatures vary substantially across these regions, generating differential selective pressures on heat tolerance: GD and HN frequently experience maximum temperatures of 38 to 42 °C, GX peaks at 34 to 36 °C, and high-elevation YN rarely exceeds 35 to 36 °C. These geographically dependent extremes create a natural gradient for examining population-level thermal adaptation and its potential evolutionary consequences [21,22].

Despite its agricultural significance, little is known concerning variation in upper thermal limits, reproductive performance following heat stress, and cross-generational responses to thermal exposure among geographically distinct populations of *D. neobrevipes*. Such information is critical for understanding the adaptive capacity of *D. neobrevipes* and predicting population responses to future climate warming. In this study, we investigate thermal tolerance across four geographically distinct Chinese populations of *D. neobrevipes* by exposing individuals to controlled acute heat stress and quantifying their survival, development, and reproductive performance, including potential effects on their offspring. We further evaluate how local climatic regimes including activity-season temperatures, episodic high-temperature exposure, and historical climatic patterns shape population divergence in thermal physiology. The findings provide insight into the evolution of heat tolerance and cross-generational thermal resilience in a major invasive agricultural pest. This will improve population predictions concerning its potential expansion under changing climate conditions and inform region-specific pest management strategy development.

## 2. Materials and Methods

### 2.1. Biological Materials

Four geographically distinct populations of *D. neobrevipes* were collected from sisal plants (*A. sisalana*) in June 2018.

Sampling sites included Leizhou City, Guangdong Province (GD; 110.10° E, 20.91° N, 13–30 m a.s.l.); Pubei County, Guangxi Province (GX; 109.56° E, 22.27° N, 34 m a.s.l.); Changjiang County, Hainan Province (HN; 109.05° E, 19.26° N, 5–30 m a.s.l.); and Jinghong City, Yunnan Province (YN; 100.80° E, 22.01° N, 558 m a.s.l.) across southern China. Mean annual temperatures ranged from 18 to 23 °C in GX, from 20 to 24 °C in GD, from 21 to 25 °C in HN, and from 19 to 23 °C in inland YN, with narrower thermal amplitude at coastal sites (GD, HN) and broader thermal amplitude at the plateau site (YN); thus providing a natural gradient for examining population-level thermal adaptation.

Following collection, all populations were reared separately on *A. sisalana* under controlled conditions (26 ± 0.5 °C, 75 ± 5% RH, 14: 10 h light-dark photoperiod) in the laboratory of Guangxi Academy of Agricultural Sciences, Nanning, China for five generations prior to experimentation. Large colony sizes (>500 individuals per generation) were maintained to minimize genetic drift and unintended selection. Laboratory rearing reduced field-acquired maternal and environmental effects while preserving population-level thermal differentiation.

### 2.2. Thermal Stress Treatments and Demographic Assays

Thermal bioassays were conducted following the method of Qin et al. [20], with modifications to accommodate the biology of *D. neobrevipes*. Newly molted adult females and newly emerged adult males from each geographic population were distinguished based on established morphological criteria; females are apterous, oval, and covered with the typical mealybug wax, whereas males are slender, winged, and also possess a single pair of delicate wings with a characteristic caudal filament. Individuals (either male or female) were placed in 90 mm Petri dishes and subjected to short-term thermal stress at 35, 38, 41, or 44 ± 0.5 °C for 2 h using precision-controlled incubators (RXZ-280B-LED, Jiangnan Instrument Co., Ltd., Ningbo, China). A temperature of 26 ± 0.5 °C served as a control. Adult survival was assessed immediately following exposure under a stereomicroscope (SMZ445, Nikon Corporation, Tokyo, Japan), with individuals displaying coordinated movement and normal posture being classified as survivors. Although survival was qualitatively monitored across all populations and temperature treatments, survival rates were not systematically quantified or statistically analyzed for all population × temperature combinations. Subsequent reproductive and F1 assays were conducted exclusively on surviving adults and therefore reflect post-stress performance conditioning on survival.

Surviving adults were transferred to controlled climatic chambers maintained at 26 ± 0.5 °C, 75 ± 5% RH, and a 14:10 h light–dark photoperiod. Fresh *A. sisalana* leaves were surface-sterilized in 70% ethanol for 5 min, rinsed in distilled water, and cut into standardized leaf segments (100 × 25 mm). Each segment was placed in an open-ended glass tube (25 mm diameter × 200 mm length), into which one mating pair of *D. neobrevipes* were introduced. The tubes were subsequently sealed with Parafilm and secured with rubber bands. Each pair was maintained individually, with 30 replicates per treatment. This sample size was determined a priori to ensure reliable estimation of demographic parameters while maintaining consistent rearing conditions.

Daily observations recorded (i) the date of first nymph deposition and (ii) the number of nymphs produced every 24 h from reproduction onset until female death. Noting that *D. neobrevipes* females lay eggs that are embedded in a waxy or cottony ovisac, that is difficult to monitor directly; the emergence of first-instar nymphs was used as a practical proxy for the onset of reproduction. Host material was refreshed every 7–10 days, and the insects were transferred carefully with a soft camel-hair brush. Nymphs from each female were transferred to ventilated plastic rearing boxes (200 × 100 × 50 mm) with fresh *A. sisalana* leaves. Once they reached the second instar, more than 50 nymphs per replicate were randomly collected, and F1 survival and the sex ratio were assessed based on morphological criteria. Each temperature × population treatment included 10 independent replicates. Sex ratio estimates were therefore based on individuals surviving to the second instar or later stages, when sex could be reliably determined morphologically.

### 2.3. Statistical Analyses

All statistical analyses were conducted in R version 4.3.2 (R Foundation for Statistical Computing, Vienna, Austria).

Pre-oviposition period, longevity, and fecundity were treated as count data and analyzed using generalized linear models (GLMs) with a log link. A Poisson model was initially fitted for each trait, and overdispersion was assessed using Pearson residual dispersion (φ). When overdispersion was detected (e.g., longevity: φ = 4.12), negative binomial GLMs were applied. Type II likelihood ratio χ^2^ tests were used to evaluate the effects of population (Location), temperature (Temperature), and their interaction.

Survival rate and sex ratio were analyzed using beta regression models with a logit link function. Proportion data were adjusted to lie within the open interval (0, 1) prior to analysis. Precision structure (φ) was evaluated by comparing constant- and varying-precision models using likelihood ratio tests, and the model with better fit was selected.

Estimated marginal means (EMMs) and their 95% confidence intervals were calculated using the emmeans package. Pairwise comparisons were conducted with adjustment for multiple testing. Statistical differences among treatments were indicated using compact letter displays.

Model fit was evaluated using simulation-based residual diagnostics (DHARMa package version 0.4.7), and no substantial deviations from model assumptions were detected.

## 3. Results

### 3.1. Pre-Oviposition Responses to Acute Heat Stress

**Figure 1:**
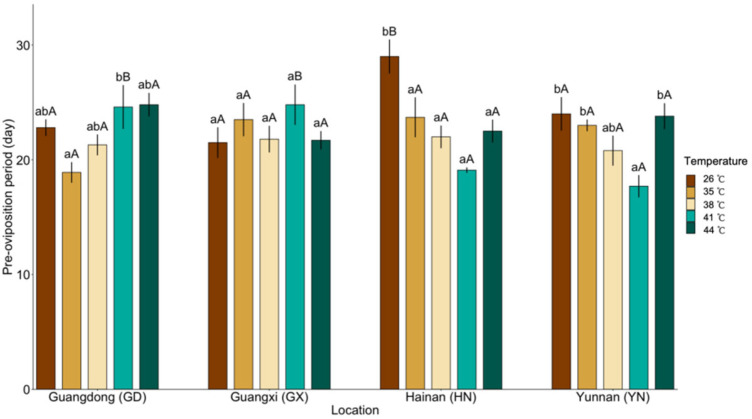
Mean (±95% CI) pre-oviposition period of *Dysmicoccus neobrevipes* from four Chinese geographic populations—Guangdong (GD), Guangxi (GX), Hainan (HN), and Yunnan (YN)—subjected to thermal stress at 35, 38, 41 and 44 °C, with 26 °C as control. Lowercase letters indicate significant differences among temperatures within each population, and uppercase letters indicate significant differences among populations within each temperature based on fitted statistical models with multiplicity-adjusted pairwise comparisons (*p* < 0.05).

The pre-oviposition period responded primarily to thermal variation rather than population identity, with a significant effect of temperature but no overall population effect (population: χ^2^ (3) = 7.48, *p* = 0.058; temperature: χ^2^ (4) = 19.09, *p <* 0.001) (Figure 1). In contrast, the population × temperature interaction was highly significant (χ^2^ (12) = 78.91, *p* < 0.001), revealing distinct population-specific thermal response patterns.

Across three populations (GD, HN, and YN), pre-oviposition period exhibited a nonlinear, U-shaped relationship with temperature, with shorter durations under intermediate heat treatments (35–38 °C) compared with both the control temperature (26 °C) and the highest temperature (44 °C) (Figure 1). Among these populations, the HN insects exhibited the strongest temperature sensitivity, displaying significantly shorter pre-oviposition periods than the control across all heat treatments (26 °C vs. 35 °C: z = 3.51, *p* = 0.004; 26 °C vs. 38 °C: z = 4.73 *p* < 0.001; 26 °C vs. 41 °C: z = 5.71, *p* < 0.001; 26 °C vs. 44 °C: z = 4.42 *p* < 0.001). In contrast, the GX population displayed no significant temperature-dependent variation in pre-oviposition period.

### 3.2. Effects of Heat Stress on Longevity

**Figure 2:**
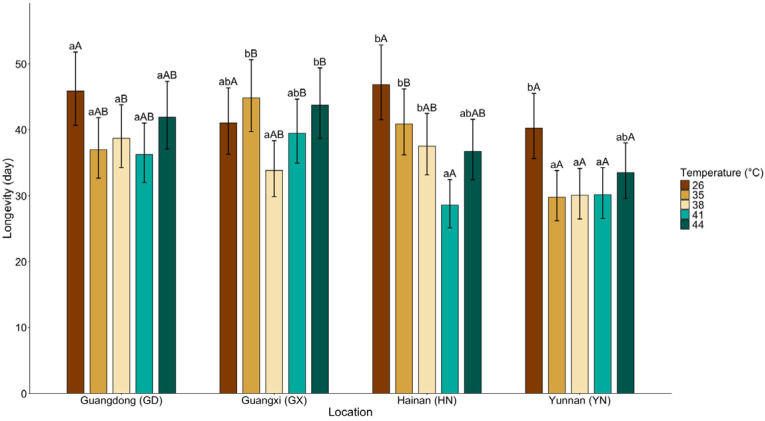
Mean (±95% CI) longevity of *Dysmicoccus neobrevipes* from four Chinese geographic populations—Guangdong (GD), Guangxi (GX), Hainan (HN), and Yunnan (YN)—subjected to thermal stress at 35, 38, 41, and 44 °C, with 26 °C as control. Lowercase letters indicate significant differences among temperatures within each population, and uppercase letters indicate significant differences among populations within each temperature based on fitted statistical models with multiplicity-adjusted pairwise comparisons (*p* < 0.05).

Female longevity exhibited pronounced population-specific sensitivity to thermal stress, as reflected by significant effects of population, temperature, and their interaction (population: χ^2^ (3) = 35.84, *p* < 0.001; temperature: χ^2^ (4) = 41.95, *p* < 0.001; population × temperature: χ^2^ (12) = 29.10, *p* = 0.004; Figure 2).

Consistent with the significant interaction, temperature effects differed among populations. In GD, longevity tended to decline under elevated temperatures relative to 26 °C, although no multiplicity-adjusted pairwise contrast reached statistical significance (e.g., 26 vs. 41 °C: z = 2.67, *p* = 0.073). In GX, longevity was significantly higher at 35 °C than at 38 °C (z = 3.17, *p* = 0.015) and higher at 44 °C than at 38 °C (z = −2.88, *p* = 0.039), whereas comparisons relative to 26 °C were not significant (all *p* ≥ 0.133). In HN, longevity declined markedly at 41 °C compared with 26 °C (z = 5.51, *p* < 0.001), 35 °C (z = 3.97, *p* < 0.001), and 38 °C (z = 3.01, *p* = 0.026). In YN, longevity at 26 °C was significantly greater than at 35 °C (z = 3.35, *p* = 0.008), 38 °C (z = 3.24, *p* = 0.012), and 41 °C (z = 3.21, *p* = 0.013), whereas the comparison between 26 °C and 44 °C was not significant (z = 2.05, *p* = 0.341). Overall, longevity responses were nonlinear and population-dependent, with the strongest reductions occurring at 41 °C in HN and at 35–41 °C in YN.

### 3.3. Population-Specific Fecundity Responses

**Figure 3:**
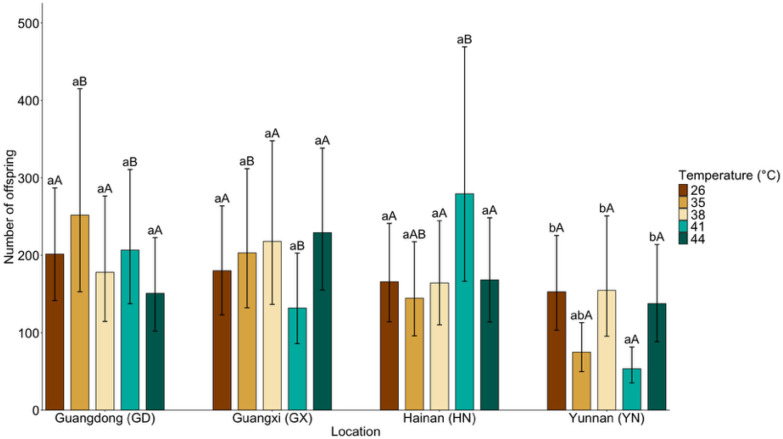
Mean (±95% CI) number of offspring of *Dysmicoccus neobrevipes* from four Chinese geographic populations—Guangdong (GD), Guangxi (GX), Hainan (HN), and Yunnan (YN)—subjected to thermal stress at 35, 38, 41, and 44 °C, with 26 °C as control. Lowercase letters indicate significant differences among temperatures within each population, and uppercase letters indicate significant differences among populations within each temperature based on fitted statistical models with multiplicity-adjusted pairwise comparisons (*p* < 0.05).

Fecundity patterns differed markedly among populations while showing no consistent response to temperature (population: χ^2^ (3) = 20.75, *p* < 0.001; temperature: χ^2^ (4) = 2.19, *p* = 0.701). Nevertheless, a significant population × temperature interaction was detected (χ^2^ (12) = 27.47, *p* = 0.007), indicating that thermal effects on fecundity were contingent on population identity (Figure 3).

Within GD, GX, and HN populations, no significant temperature effects were detected (all adjusted *p* > 0.05), although mean fecundity in HN was numerically higher at 41 °C (41 vs. 26 °C: z = −1.60, *p* = 0.498). In contrast, the YN population exhibited a pronounced decline at 41 °C. Fecundity at 41 °C was significantly lower than at 26 °C (z = 3.58, *p* = 0.003) and 38 °C (z = 3.25, *p* = 0.010), and differed significantly from 44 °C (z = −3.03, *p* = 0.020). Across populations, significant differences were primarily detected at 41 °C, where HN (z = 4.850, *p* < 0.001), GX (z = 2.94, *p* = 0.017), and GD (z = 4.52, *p* < 0.001) all exhibited higher fecundity than YN. These results indicate that fecundity variation under heat stress was largely driven by the strong decline observed in YN at 41 °C. Interestingly, female longevity in the GD, HN, and YN populations followed a U-shaped response to temperature (Figure 2), assisting in explaining the observed population-specific decoupling between fecundity and lifespan under heat stress.

### 3.4. Cross-Generational Effects on Sex Ratio and Survival

**Figure 4:**
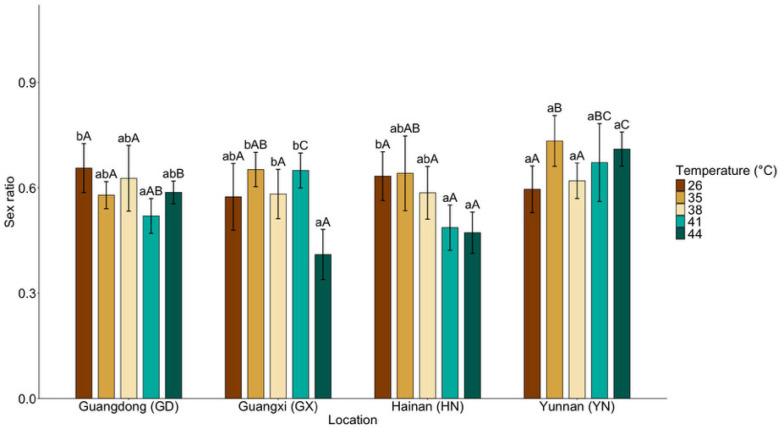
Mean (±95% CI) sex ratios of the F1 generation of *Dysmicoccus neobrevipes* from four Chinese geographic populations—Guangdong (GD), Guangxi (GX), Hainan (HN), and Yunnan (YN)—subjected to thermal stress at 35, 38, 41, and 44 °C, with 26 °C as control. Lowercase letters indicate significant differences among temperatures within each population, and uppercase letters indicate significant differences among populations within each temperature based on fitted statistical models with multiplicity-adjusted pairwise comparisons (*p* < 0.05).

Cross-generational traits exhibited strong population-dependent responses to thermal stress, with significant effects of population, temperature, and their interaction on F1 sex ratios (population: χ^2^ (3) = 63.28, *p* < 0.001; temperature: χ^2^ (4) = 56.25, *p* < 0.001; population × temperature: χ^2^ (12) = 50.96, *p* < 0.001; Figure 4).

Within populations, temperature effects differed in magnitude. In the GX population, sex ratios reached their minimum at 44 °C, with a significant overall thermal effect (χ^2^ (4) = 22.65, *p* < 0.001). The GD population showed its lowest sex ratio at 41 °C, and the overall temperature effect within GD was also significant (χ^2^ (4) = 10.29, *p* = 0.036). The HN population exhibited a progressive decline in sex ratio with increasing temperature, with a significant overall thermal effect (χ^2^ (4) = 14.37, *p* = 0.006). In contrast, the YN population showed a peak sex ratio at 35 °C and no significant temperature-dependent variation at higher temperatures (χ^2^ (4) = 8.87, *p* = 0.065).

**Figure 5:**
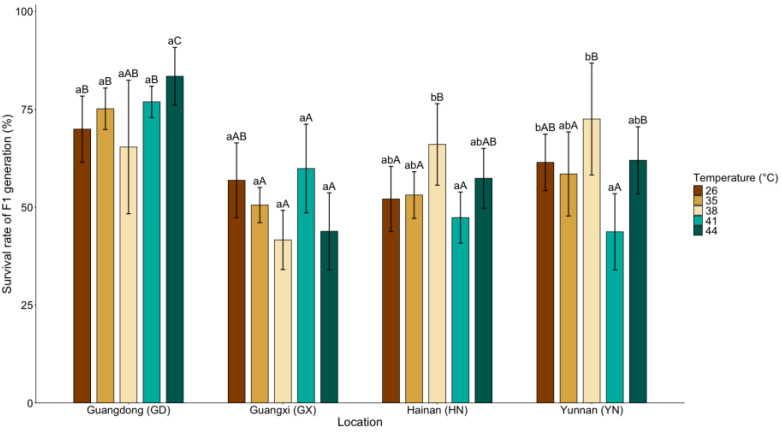
Mean (±95% CI) survival rate of the F1 generation of *Dysmicoccus neobrevipes* from four Chinese geographic populations—Guangdong (GD), Guangxi (GX), Hainan (HN), and Yunnan (YN)—subjected to thermal stress at 35, 38, 41, and 44 °C, with 26 °C as control. Lowercase letters indicate significant differences among temperatures within each population, and uppercase letters indicate significant differences among populations within each temperature based on fitted statistical models with multiplicity-adjusted pairwise comparisons (*p* < 0.05).

A broadly similar population-dependent pattern was observed for F1 survival (Figure 5). Survival was strongly structured by population identity and was also significantly influenced by temperature (population: χ^2^ (3) = 91.95, *p* < 0.001; temperature: χ^2^ (4) = 34.31, *p* = 0.005). The significant population × temperature interaction (χ^2^ (12) = 32.36, *p* = 0.001) further indicates population-specific sensitivity of offspring survival to thermal stress.

Within populations, temperature effects differed in magnitude. In the GD population, F1 survival remained relatively stable across temperature treatments, and no significant overall temperature effect was detected (χ^2^ (4) = 3.78, *p* = 0.437). In the HN population, F1 survival showed a significant overall response to temperature (χ^2^ (4) = 11.85, *p* = 0.019). Post hoc comparisons revealed a significant difference between 38 °C and 41 °C (z = 2.98, *p* = 0.028), whereas other pairwise contrasts were not significant. Similarly, the GX population exhibited a significant overall temperature effect (χ^2^ (4) = 10.91, *p* = 0.028), although no individual temperature contrast reached statistical significance, with survival tending to be lower at 38 °C and 44 °C. The YN population exhibited the strongest temperature-dependent variation (χ^2^ (4) = 16.45, *p* = 0.002), consistent with the marked decline in survival observed at 41 °C.

Across temperatures, significant population differences were detected at all thermal treatments, with the strongest divergence observed at 44 °C (χ^2^ (3) = 31.68, *p* < 0.001), where the GD population maintained the highest survival, supporting its superior thermal tolerance.

Collectively, these results show that acute heat stress drives strong population-specific responses across life-history and cross-generational traits, underscoring the role of divergent thermal tolerance and allocation strategies in shaping demographic resilience.

## 4. Discussion

Geographic variation in thermal environments has driven pronounced population-level divergence in upper thermal performance limit and heat resilience in *D. neobrevipes*. Our findings demonstrate clear geographic divergence within upper thermal limits, with populations from Guangdong (GD) and Hainan (HN) exhibiting consistently higher heat tolerance, survival, and reproductive stability under acute high-temperature stress than populations from Guangxi (GX) and Yunnan (YN). This pattern is consistent with the long-term exposure of the GD and HN populations to warmer activity-season temperatures and recurrent summer heat extremes [18,19]. Across insect taxa, populations inhabiting warmer or more thermally variable environments frequently evolve broader thermal tolerance and stronger heat-shock resistance, providing a comparative context for our findings [13,23]. By contrast, the GX and especially YN populations, which originated from cooler regions with fewer extreme heat events, showed markedly lower upper thermal limits. Such divergence aligns with evolutionary expectations that chronic exposure to higher temperatures strengthens physiological protection systems including heat shock response efficiency [4,24], antioxidant defenses [25], and membrane restructuring [5]. In comparison, cooler climates impose weaker and less consistent directional selection on upper thermal limits, despite potentially severe short-term fitness costs during rare heat events [15,26]. Similar geographic differentiation in thermal sensitivity has been documented across diverse insect taxa that are experiencing climate warming and increasing thermal extremes [27].

Adult performance across populations followed a characteristic nonlinear thermal performance curve, with short-term benefits under moderate heat and rapid collapse beyond upper thermal limits. Across multiple life-history traits, moderate heat exposure within the typical activity-season range (35–38 °C) maintained or even enhanced adult performance, likely reflecting accelerated metabolism, mild stress priming, and activation of protective cellular processes such as antioxidant and detoxification pathways [3,7]. Experimental studies in insects such as *Drosophila melanogaster* Meigen, 1830 (Diptera: Drosophilidae), *Tribolium castaneum* Herbst, 1797 (Coleoptera: Tenebrionidae), and *Manduca sexta* Linnaeus, 1763 (Lepidoptera: Sphingidae) have similarly demonstrated that sublethal heat exposure can transiently enhance heat resistance and short-term performance [12,28,29,30]. Consistent with this pattern, our previous work on *D. neobrevipes* demonstrated increased antioxidant enzyme activity (SOD, CAT, and POD) under moderate heat stress [25]. However, once temperatures exceeded population-specific upper thermal limits (≥41 °C), adult longevity, fecundity, offspring survival, and sex ratio stability declined sharply [4,6,16]. These threshold-driven collapses indicate that even small increases beyond critical temperatures can synchronously impair multiple fitness-related traits, explaining why GD maintained performance under extreme heat, whereas HN showed partial resilience but significant reductions in specific traits [3]. Such abrupt breakdowns above thermal thresholds are increasingly recognized as key determinants of insect population persistence under extreme temperature events [15,31].

The populations differed markedly in life-history strategies under heat stress, revealing contrasting trade-offs between survival and reproduction shaped by thermal history [32,33]. The HN population exhibited a pronounced stress-induced trade-off: despite reduced longevity at 41 °C, fecundity did not decline and was numerically higher, suggesting a potential shift in allocation rather than a statistically confirmed increase. This is consistent with terminal investment theory whereby reproductive effort is elevated when potential future prospects decline [34]. In contrast, the YN population showed uniformly poor performance across traits, lacking evidence of compensatory reproductive responses. These differences likely reflect contrasting thermal histories, as repeated exposure to high summer temperatures can favor both compensatory reproductive strategies and enhanced physiological stability, whereas populations from cooler regions experience weaker selective filtering for such responses [7,11,20,26,35]. This interpretation is supported by broader evolutionary and physiological evidence showing that thermal selection can drive divergence in metabolic allocation, stress tolerance, and population-level variation in acclimation capability [36,37,38].

These patterns are consistent with carry-over effects associated with parental conditioning and selective survival. However, adaptive transgenerational mechanisms cannot be conclusively excluded. Variations in offspring survival and sex ratio following parental heat exposure closely mirrored adult thermal tolerance across populations, indicating strong carry-over effects from parental conditioning. As only adults surviving acute heat exposure contributed to fecundity and F1 assays, offspring outcomes inevitably incorporate a survivorship filter favoring more heat-tolerant individuals. Such survivorship bias is intrinsic to acute stress experiments and can exaggerate apparent tolerance or reproductive stability by disproportionately retaining individuals with greater stress resistance [39,40]. Within this context, population-specific offspring responses likely arise through two non-exclusive short-term processes. Acute heat exposure may induce reversible physiological shifts in surviving adults, altering reproductive allocation or gamete provisioning, consistent with terminal investment theory [33]. Simultaneously, strong viability selection during heat stress may result in surviving parents representing a non-random subset with intrinsically higher thermal tolerance, producing offspring with enhanced performance without invoking adaptive transgenerational plasticity. Accordingly, the observed responses of the offspring are best interpreted as apparent carry-over effects shaped by parental conditioning and selective survival rather than definitive evidence of adaptive transgenerational plasticity [4,6,41,42].

Under increasingly frequent periods of extreme heat, population-specific thermal limits and life-history trade-offs are likely to play a decisive role in shaping pest persistence and outbreak risk. By integrating adult performance, life-history trade-offs, and cross-generational responses, our findings demonstrate that population divergence in thermal tolerance arises from the combined effects of climatic adaptation, nonlinear physiological limits, and allocation trade-offs. This interpretation is further supported by previous experimental evidence from the Guangxi population of *D. neobrevipes*, which showed that adult survival declined significantly only at extreme temperatures (44 °C) during early adult stages, whereas moderate heat treatments caused little or no mortality at later stages [43]. Such population-specific thermal thresholds suggest that the demographic consequences of heat stress may remain cryptic until critical limits are exceeded, reinforcing the importance of extreme events rather than gradual warming alone. Although all populations were maintained under identical laboratory conditions for no more than five generations prior to experimentation and population sizes were sufficiently large to minimize genetic drift, we acknowledge that short-term laboratory rearing may still influence thermal traits through plastic or adaptive responses. Nonetheless, the persistence of clear population-specific thermal responses suggests that underlying differences were not fully eroded, although caution is warranted when extrapolating laboratory estimates to natural populations. As extreme heat events increasingly dominate thermal variability in subtropical agroecosystems, such population-specific constraints are likely to outweigh gradual warming in determining demographic resilience [44,45].

Understanding how invasive and economically important pests respond to extreme heat is therefore essential for predicting future outbreak dynamics, geographic spread, and invasion risk [7,46,47]. Consistent with broader evidence that climate warming and extreme events can strongly alter population dynamics and species interactions [48,49], our results highlight the need for future studies integrating genomic, biochemical, and fine-scale field temperature data to assess how laboratory patterns translate into long-term persistence and spread [19,50,51]. Ultimately, clarifying how populations respond to thermal extremes will be central to predicting shifts in insect life histories and fitness trajectories when experiencing climate change [38,52].

## Data Availability

The data presented in this study is available upon reasonable request from the corresponding authors. The data are not publicly available due to privacy.
